# Assessment of the dentine microhardness following the application of different intracanal medicaments. An in-vitro study

**DOI:** 10.1038/s41405-026-00408-1

**Published:** 2026-03-17

**Authors:** Sara Gamal Elgamal, Kareem Hamdy Ahmed Aly, Nadia Saeed Hosny

**Affiliations:** 1 Endodontic department, Faculty of Dentistry, Cairo university, Cairo, Egypt; 2 Conservative dentistry department, Faculty of Dentistry, Fayoum university, Fayoum, Egypt

**Keywords:** Root canal treatment, Dental biomaterials

## Abstract

**Background:**

Intracanal medications used during routine endodontic treatment and regenerative endodontic procedures might cause undesirable effects on tooth properties, such as a reduction in dentine microhardness.

**Objectives:**

To compare the effect of five intracanal medicaments; Bio-C Temp (Angelus, Brazil), modified triple antibiotic electrospun nanofibers (m-TAP) (NanoEbers, Egypt), Levofloxacin (Memphis, Egypt), Calcium hydroxide (CH) (Meta biomed, Korea) and Simvastatin (Merck & Co., USA) on the microhardness of radicular dentine.

**Methods:**

Forty-five extracted single-rooted teeth were decoronated to a 15 mm root length then prepared. The specimens were assigned to five groups according to the used intracanal medicament: Bio-C Temp, m-TAP nanofibers, Levofloxacin, CH, and Simvastatin. After medicament application for two weeks, dentine microhardness was measured using a Vickers microhardness tester. Data were analyzed using the Kruskal–Wallis and Mann–Whitney *U* tests with a significance level set at *p* < 0.05.

**Results:**

Bio-C Temp, and m-TAP nanofibers recorded the highest overall dentine microhardness values, followed by Levofloxacin and Simvastatin, while CH showed the lowest. Significant differences were observed among the tested groups at the coronal and middle levels (*p* < *0.05*), whereas no significant difference was found apically.

**Conclusions:**

Bio-C Temp, and m-TAP nanofibers demonstrated superior ability to preserve dentine microhardness, suggesting their promising potential advantage over conventional medicaments for strengthening root dentine.

## Introduction

The long-term success of endodontic treatment, particularly in regenerative endodontics, is thought to depend on the total eradication of microbial pathogens from the root canal system and the maintenance of the structural integrity of the root dentine [1]. Such a decrease in dentine microhardness (MH) may weaken the tooth’s structure and increase its vulnerability to fracture, which could lead to treatment failure or even tooth loss [2]. Therefore, improving treatment protocols and guaranteeing predictable results require an understanding of the potential effects of chemical irrigants and medicaments on the mechanical properties of root dentine [3].

Most of the irritants are eliminated by chemomechanical preparation; however, complete debridement is still difficult because of the intricate structure of root canals [4]. As far as we are aware, no single irrigant can completely clean and sterilise the canal, remove both organic and inorganic materials, maintain the integrity of the root dentine, and have no cytotoxic impact at high concentrations. Thus, applying intracanal medications is a crucial part of the disinfection procedure in endodontic therapy to improve bacteria reduction [5].

Calcium hydroxide (CH) is the most extensively used intracanal medicament in endodontics. CH’s chemical kinetics, as revealed by ionic dissociation into calcium and hydroxyl ions, characterizes its biological actions. The hydroxyl ions demonstrate bactericidal effects, including the rupture of bacterial cell membranes, protein denaturation, and DNA damage [6]. Furthermore, they enhance tissue repair by neutralizing the low pH environment caused by inflammation. Furthermore, it stimulates the alkaline phosphatase enzyme, which promotes mineralized tissue development and periapical healing [7].

Despite its potential benefits as an intracanal medication, CH has certain drawbacks, including decreased antimicrobial efficacy against certain microorganisms, the buffering effect of dentine at high pH levels caused by CH, a delayed onset of action, and challenges with removal [8]. According to studies, long-term usage of CH can impair tooth fracture resistance because of its high alkalinity and water solubility, which cause dentine’s organic and inorganic components to deteriorate [9].

The quest for intracanal drugs that successfully stop bacterial development and enhance tissue regeneration has intensified in order to get beyond the forementioned restrictions. Recently, Bio-C Temp, a revolutionary pre-mixed, ready-to-use intracanal medication based on calcium silicate, was released. It is made up of calcium silicate, calcium aluminate, calcium oxide, a base resin, and radiopacifying substances including calcium tungstate and titanium oxide [10]. Bio-C Temp is biocompatible over time and does not inhibit the release of dentine-derived growth factors, such as transforming growth factor beta 1 [11]. Along with its established antibacterial effect, makes it a viable candidate for regenerative endodontic therapies [12]. Bio-C Temp was found to have no detrimental effects on dentine mechanical properties, including microhardness and push-out bond strength [10].

Electrospinning is an innovative technology in nanofiber fabrication using a high-voltage electric force to create nanofibers out of polymer solutions [13]. Electrospinning technology has been used in regenerative endodontics to fabricate drug loaded polymer-based nanofibers with the minimum efficient concentration [14]. Previous studies have showen that electrospun nanofibers containing antibiotic had higher antibacterial activity when compared to other intracanal medicaments [15–17].

Levofloxacin is a third-generation, broad-spectrum fluoroquinolone antibiotic that is used in many different medical specialties and has demonstrated significant effectiveness in preventing the formation of biofilms. Levofloxacin has better bioavailability, more reliable absorption, and increased bactericidal potency—especially against Enterococcus faecalis—than Ciprofloxacin [18]. It has a prolonged post-antibiotic impact and is two to four times more active against Gram-positive bacteria, suggesting that it can prevent bacterial growth even after removal [19]. Additionally, it is better suitable for use in regenerative endodontic procedures  due to its lower minimum inhibitory concentration (MIC) [20, 21].

Statins are safe and efficient drugs that lower the liver’s production of cholesterol [22]. Because of their many biological characteristics, including anti-inflammatory, antibacterial, and osteogenic effects, as well as their ability to promote dentineogenesis, statins have drawn interest in the field of endodontics [23]. Research has demonstrated that statins can increase the release of bioactive molecules from the dentine matrix, which is crucial for encouraging dentine repair and regeneration [24]. The mineralization-promoting effect of Simvastatin may contribute to the long-term preservation or perhaps improvement of dentine integrity [25]. Furthermore, a previous study [26] has demonstrated that, when compared to CH and chlorohexidine as intracanal medications, Simvastatin preserves or enhances fracture resistance with extended administration.

To our knowledge, no study has investigated the effect of Bio-C Temp, pre-synthetized modified triple antibiotic electrospun nanofibers (m-TAP) nanofibers, Levofloxacin, CH and Simvastatin on root dentine microhardness. Therefore, this study aimed to determine the effect of these five intracanal medicaments on root dentine microhardness. The null hypothesis is that there is no difference between the efficacy of Bio-C Temp, m-TAP nanofibers, Levofloxacin, CH and Simvastatin as intracanal medicaments on root dentine microhardness.

### Sample size

A sample size of forty-five teeth (9 per group) was selected. The sample size was calculated using G*Power 3.1 software. Based on a previously published study [10] that reported an effect size (Cohen’s f) of approximately 2.17 for dentine microhardness differences among intracanal medicaments, and assuming a significance level of 0.05 and power of 80%.

## Materials and methods

**Fig. 1:**
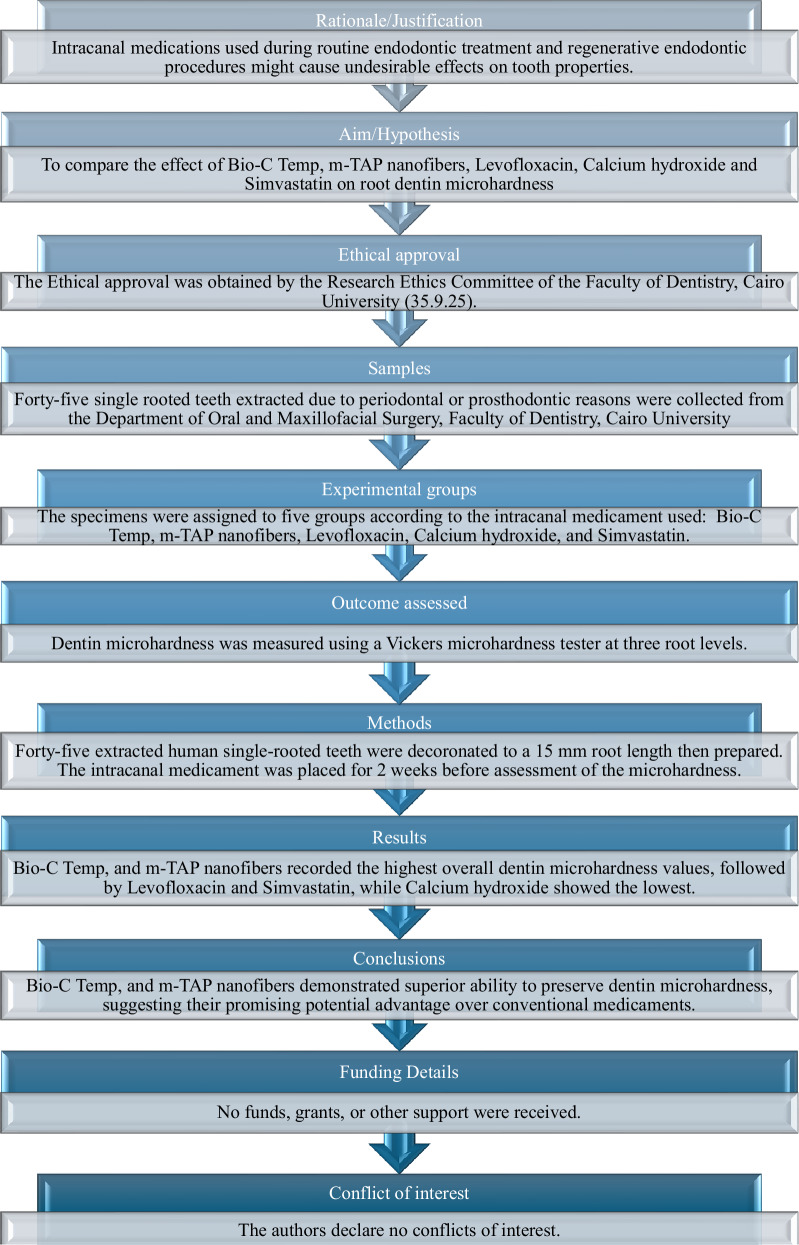
PRILE 2021 flowchart of the study.

The materials used in this study are described in Table 1. A PRILE flow chart is presented in Fig.1. Forty-five single-rooted teeth, extracted due to periodontal or prosthodontic reasons in patients aged 18–40 years, were collected from the Department of Oral and Maxillofacial Surgery, Faculty of Dentistry, Cairo University. In order to assess the internal anatomy and verify that the eligibility requirements are met without any complications or flaws, multiple angulation pre-operative radiographs were taken. Teeth with a single canal, a closed apex, and no indications of internal resorption, calcification, or prior endodontic therapy were included.

**Table 1:** Materials used in the study

Material	Specification	Manufacturer /preparation and composition
Bio- C Temp	Tricalcium silicate ready to use paste for intracanal dressing	Angelus, Londrina, PR, Brazil (calcium silicate, calcium oxide, zirconium oxide, thickening agents)
m-TAP nanofibers	Electrospun nanofibers containing Ciprofloxacin, Metronidazole and Clindamycin at a concentration of 0.1 mg/ml	335 mg of each of the 3 drugs were added to a polymer solution of 7% polyvinyl alcohol (Oxford laboratories, India) and stirred at a concentration of 30 wt. %. The solution was then loaded to the syringe of the Electrospinning system (NanoEbers, LLC Egypt).
Levofloxacin	Fluoroquinolone antibiotic Prepared in a gel form at concentration of 16 mg/ml	80 mg of methylcellulose (Sigma-Aldrich Chemie GmbH, Schnelldorf, Germany) was added to 1 ml of distilled water. The mixture was heated to 700 C with continuous stirring and left to cool to room temperature. 16 mg Tavanic 500 mg (Memphis, Egypt) were added and stirred.
Calcium hydroxide (CH)	Calcium hydroxide ready to use temporary root canal filling material	Metapaste. Meta biomed Co., Chungbuk-do, Korea (Calcium hydroxide, barium sulphate, purified water and polypropelene glycol, thickening agents)
Simvastatin	Antilipidemic drug of the statin class Prepared in a gel form at concentration of 10 mg/ml	80 mg of methylcellulose (Sigma-Aldrich Chemie GmbH, Schnelldorf, Germany) was added to 1 ml of distilled water. The solution mixture was heated to 700 C with continuous stirring and left to cool to room temperature, then 10 mg of Zocor (Merck & Co., Inc, white house station, N.J., USA) were added and stirred.

### Preparation of sample

After using a curette to clean the teeth’s exterior root surface, teeth were sterilised using sodium hypochlorite (Vishal Dentocare Pvt. Ltd., Ahmedabad, India) solution and an ultrasonic cleaner (Codyson, China). The teeth were then stored in saline solution (ADWIC, Cairo, Egypt) until use. To achieve a typical root length of 15 mm, clinical crowns were sectioned at the cementoenamel junction. The working length of each root was determined by inserting a size #10 K-file (Dentsply Maillefer, Switzerland) into the root canal until the tip was visible in the apical foramen. The file was then pulled back one millimeter under magnification. ProTaper Gold rotary files (Dentsply Maillefer, Ballaigues, Switzerland) up to size F4 were used to prepare the canals at a speed of 250 rpm and 3 Ncm torque, according to the manufacturer’s instructions. Each file was used to prepare a maximum of 4 canals to ensure safe and effective canal preparation. Shaping files(SX, S1and S2) were used in a brushing motion, while finishing files (F1–F4) were used in a non-brushing action till reaching the full working length. A side-vented needle gauge 30 (Kerr SA, Bioggio, Switzerland) 1 mm shorter than the working length was used for the irrigation. Between each file, 3 mL of freshly made 2.5% sodium hypochlorite solution was used to irrigate the canals for one minute along with short vertical strokes of manual dynamic activation [27]. Sterile paper points (Meta Biomed Co., Chungcheongbuk-do, Korea) were used to dry the teeth after a 3 ml saline final rinse.

The samples were then randomly divided into 5 groups (*n* = 9), according to the intracanal medicaments used, as follows Group 1; Bio-C Temp, Group 2; m-TAP nanofibers, Group 3 Levofloxacin, Group 4; CH and Group 5; Simvastatin. Intracanal medicaments were incrementally placed into the root canals using direct syringe injection approach that was carefully withdrawn coronally until backfill at the canal orifice was observed. The coronal portion of the root canal was sealed with a temporary filling material (Orafil-G; Prevest DenPro, India). Teeth were kept in an incubator (Binder, York, UK) at 37 °C and 100% humidity for two weeks. The medications were then removed using the last used instrument along with 20 ml of sterile saline irrigation [9]. Teeth were embedded in auto-polymerized acrylic resin blocks then sectioned longitudinally using a diamond disc (0.6 mm) and a precision saw (Isomet, Buehler, USA) at 2500 rpm, under copious irrigation.

### Assessment of radicular microhardness

Three sections of each tooth were used to measure the microhardness. The specimens were polished using a polisher machine (APL-4, Arotec Ind., Brazil) and carbide abrasive papers of increasing granulation (#320 #600 and #1200, Bego, Germany) for 1 min, to obtain a smooth glossy surface. Teeth were then cleaned again using the ultrasonic cleaner. The radicular dentine surface hardness measurement was carried out using Vickers Diamond Microhardness Tester (Wilson Microhardness Tester model TUKON 1102, Germany). The indenter was forced into the test specimen, at 1 mm from the root canal wall, by a smooth, impact-free application of a 25-g weight. The indenter was kept in place for 10 s. Following the removal of the load, the indentation was focused using the magnifying eye piece, and the two impression diagonals were measured with a micrometer to the closest 0.1 μm, then averaged [10].

### Statistical analysis

Data were tested for normality using the Shapiro–Wilk test and were found to be non-parametric. Therefore, intergroup comparisons were performed using the Kruskal–Wallis test, followed by pairwise comparisons with the Mann–Whitney *U* test adjusted using the Bonferroni correction. Data are presented as either mean ± standard deviation (SD) or median and interquartile range (IQR), as appropriate. A significance level of *p* < 0.05 was adopted for all tests. Statistical analyses were performed using SPSS software (Version 29.0; IBM Corp., Armonk, NY, USA).

### Ethics declaration

The present study was carried out following approval by the Research Ethics Committee of the Faculty of Dentistry, Cairo University (35.9.25).

## Results

**Fig. 2:**
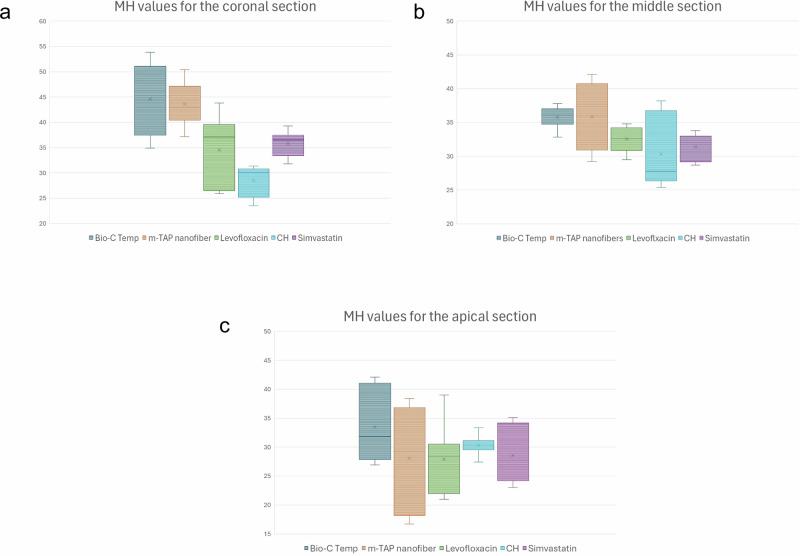
Box plots for comparison of dentine microhardness (MH) among the five tested intracanal medicaments at three root levels. **a** MH values for the coronal root section, **b** MH values for the middle root section, and **c** MH values for the apical root section.

At the coronal level, Bio-C Temp and m-TAP nanofibers groups demonstrated the highest microhardness values, showing significantly greater mean and median values compared to CH (*p* < 0.001). Levofloxacin and Simvastatin exhibited intermediate values that were statistically comparable to the Bio-C Temp and m-TAP nanofibers groups but higher than CH Fig. 2A. A similar trend was observed at the middle third, where Bio-C Temp maintained the highest values, while CH showed the lowest, with a statistically significant difference among the groups (*p* = 0.008) Fig. 2B. In contrast, at the apical level, there was no significant difference among all tested medicaments (*p* = 0.331), indicating a reduced microhardness toward the apical third regardless of the material used Fig. 2C, Table 2.

**Table 2:** Inter-group comparison of the radicular dentin microhardness

	Coronal		Middle		Apical	
	Median(IQR)	Mean (SD)	Median(IQR)	Mean (SD)	Median(IQR)	Mean (SD)
*Bio-C Temp*	45.1(13.7)^a^	44.58 ± 6.87	36.1(2.25)^a^	35.79 ± 1.56	31.8 (13.2)^a^	33 ± 6.2
*m-TAP nanofiber*	43(6.7)^a^	43.6 ± 4.15	35.9(9.85)^ab^	35.83 ± 4.89	29.3 (18.65)^a^	28.1 ± 8.59
*Levofloxacin*	37.1(13.1)^ab^	34.5 ± 6.66	32.6 (3.35)^ab^	32.55 ± 1.82	28.4 (8.6)^a^	27.9 ± 5.69
*CH*	30 (5.6)^b^	28.5 ± 3.02	27.8 10.4)^b^	30.32 ± 5.26	30.3 (1.6)^a^	30.33 ± 1.62
*Simvastatin*	36.5(4.05)^ab^	35.8 ± 2.51	32 (3.85)^ab^	31.4 ± 1.95	27.7(9.95)^a^	28.54 ± 4.77
*p value*	*p* < 0.001*		*p* = 0.008*		*p* = 0.331 ns	

Different superscript letters (a, b) within the same column indicate a statistically significant difference (*p* < *0.05*, Kruskal–Wallis followed by Mann–Whitney *U* test). ns = non-significant,* significant.

*SD* standard deviation, *IQR* interquartile range.

For Bio-C Temp, dentine microhardness was highest at the coronal level and gradually decreased toward the apical third, with a significant difference among levels (*p* = 0.005). m-TAP nanofibers showed a similar trend, with significantly higher values coronally and in the middle third compared to the apical third (*p* < 0.001). In Levofloxacin, no significant difference in microhardness was found among the three levels (*p* = 0.051, ns), CH also showed no significant variation among levels (*p* = 0.486, ns). In contrast, Simvastatin showed significantly higher microhardness in the coronal third compared to the middle and apical third (*p* = 0.002). Table 3

**Table 3:** Intra-group comparison of the radicular dentin microhardness

Root Level	Calcium silicate	m-TAP Nanofibers	Levofloxacin	CH	Simvastatin
Coronal					
Median(IQR)	45.1 (13.7)	43.0 (6.7)	37.1 (13.1)	30.0 (5.6)	36.5 (4.05)
Mean (SD)	44.58 ± 6.87^a^	43.60 ± 4.15^a^	34.50 ± 6.66 ^a^	28.52 ± 3.02^a^	35.80 ± 2.51^a^
Middle					
Median(IQR)	36.1 (2.25)	35.9 (9.85)	32.6(3.35)	27.8 (10.4)/	32.0 (3.85)
Mean (SD)	35.79 ± 1.56^a^	35.83 ± 4.89^a^	32.55 ± 1.82 ^a^	30.32 ± 5.26^a^	31.40 ± 1.95^b^
Apical					
Median(IQR)	31.8 (13.2)	29.3 (18.65)	28.4 (8.6)	30.3 (1.6)	27.7 (9.95)
Mean (SD)	33.00 ± 6.20^b^	28.11 ± 8.59^b^	27.90 ± 5.69^a^	30.33 ± 1.62^a^	28.54 ± 4.77^b^
*p* Value	*p* = 0.005*	*p* < 0.001*	*p* = 0.051 ns	*p* = 0.486 ns	*p* = 0.002*

Different superscript letters (a, b) within the same column indicate a statistically significant difference (*p* < *0.05*, Kruskal–Wallis followed by Mann–Whitney *U* test). ns = non-significant,* significant.

*SD* standard deviation, *IQR* interquartile range.

## Discussion

The current study relied on the hypothesis that there would be no significant difference in the MH of canal dentine when employing Bio-C Temp, pre-synthetized m-TAP nanofibers, Levofloxacin, CH and Simvastatin as inter-appointment root canal dressings.

The microhardness of root dentine is frequently linked to its mineral content, where lower microhardness suggests demineralization and higher microhardness indicates better mineralization [28]. A decrease in root dentine microhardness results in a softer dentine structure, leading to poor adhesion and adaptation of obturation materials [29]. This compromise in sealing can negatively impact the outcome of endodontic treatments and may increase the risk of root fractures [30]. Given the challenge of preserving tooth microhardness and achieving complete or near-complete eradication of microbes within root canals, there is a necessity to identify the most effective intracanal medicament capable of addressing both aspects [31].

In this study the intracanal medicament was placed for a period of two weeks. In endodontic regeneration procedures, the duration of intracanal medicaments varies widely with an average of two to three weeks [32]. Studies have found a negative correlation between the duration of medicament placement and microhardness scores [33].

Regarding MH results, the results suggest that Bio-C Temp and m-TAP nanofibers medicaments exhibited superior microhardness values, particularly in the coronal and middle regions of the root canal, whereas CH recorded the lowest values. For the apical part of the root a non-statistically significant difference was found between the intracanal medicaments. These findings agree with a recent study [10] in which Bio-C Temp exhibited the highest MH values and CH showed the lowest values. Furthermore, other studies [20, 34, 35] also showed that CH had the lowest values for MH among all groups. However, it contradicts the results of another study [27], where CH had higher MH values compared to antibiotic, the contradiction may be attributed to the fact that in that study, Minocycline was used, which is known to chelate calcium and create insoluble complexes while in this study, instead the m-TAP nanofibers contained modified formulation with Clindamycin.

In this study, intragroup comparison showed statistically significant higher MH values in the coronal and middle root section within both the Bio-C temp and m-TAP nanofibers groups, while a non-statistically significant difference was found in the Levofloxacin and CH groups. Simvastatin showed a statistically significant difference in the coronal section as compared to both the middle and apical radicular dentine. Generally, for all groups higher MH values were observed in the coronal and middle regions and a noticeable reduction in the apical third, which might be attributed to the variations in root dentine anatomy and the amount of intracanal medicament diffusing into the apical part.

These findings are in agreement with other studies [9, 31, 36], which revealed that there was a gradual decline in microhardness mean values of root dentine along the root length. This may be attributed to the higher volume of minerals in the coronal one-third, along with anatomical variations of the number, size, and direction of dentinal tubules. This higher mineral content makes dentine more resilient and capable of resisting local deformation [37]. The tubular density of dentine has been observed to elevate as one progress from the cervical to the apical regions of radicular dentine, leading to a negative correlation between radicular dentinal microhardness and radicular tubular density [30].

Bio-C Temp facilitates maximum surface hardness, which can be elucidated by its bioactive calcium silicate composition, which releases calcium and hydroxyl ions that promote apatite nucleation and mineral deposition within dentinal tubules [10]. The material’s alkaline pH enhances the precipitation of calcium phosphate crystals, leading to increased dentine hardness [38].

One essential requirement for an intracanal medicament is its stability and sustained release over an extended period. The smallest yet effective advised antibiotic concentration 0.1 mg/ml was made possible by the usage of m-TAP in the form of nanofibers compared to the 1 g/ml in conventional paste formulation [17]. These nanofibers were previously found to have a potent antibacterial activity [13, 17, 39–41]. The acidic characteristic of antibiotics in TAP has the potential to demineralize the inorganic component of dentine, the low concentration of antibiotics may have contributed to the high MH values reported in this study, in contrast to another study that used m-TAP paste and found results comparable to CH [42].

The alkaline pH of CH contributes to the denaturation of the dentine collagenous matrix [9]. Additionally, CH modifies the three-dimensional conformation of tropo-collagen by penetrating the intrafibrillar structure of the mineralized collagen fibrils due to its low molecular weight and inorganic nature [36]. The breakdown of the connection between the hydroxyapatite crystals and collagen fibers results in a decrease in dentine microhardness [21].

Studies investigating the direct effect of Levofloxacin and Simvastatin on dentine microhardness are somehow lacking in literature. Previous investigations have reported that antibiotic-based intracanal medicaments may adversely affect dentine microhardness owing to its acidic demineralizing nature [21, 42]. In contrast, simvastatin has been shown to influence mineralization and hard-tissue formation in dental tissues, suggesting a potential impact on dentine properties [26].

The findings of this study highlight the need for clinicians to carefully select intracanal medicaments by balancing their therapeutic benefits against potential adverse effects on dentine structure, especially in cases requiring prolonged disinfection. However, this has to be seen in the light of some limitations. Despite strict inclusion criteria, standardization of extracted teeth remains challenging due to the inherent anatomical diversity. The results may not be as applicable to multi-rooted teeth with more complex canal anatomy. The inherent radiopacity of some of the tested materials limited its radiographic evaluation. Furthermore, the inclusion of a negative control group could have reinforced the interpretation of the observed effects and offered additional baseline data.

Under the limitations of this study, the null hypothesis was rejected, Bio-C Temp, and m-TAP nanofibers demonstrated superior ability to preserve dentine microhardness, suggesting their promising potential advantage over conventional medicaments for strengthening root dentine. Further studies are required to assess the long-term effects of these medicaments on dentine microhardness and their performance under aging, dynamic loading or simulated clinical conditions.

## Data Availability

The datasets of the study are available from the corresponding author on reasonable request.
